# Person-Place Fit and Quality of Life Among Older Adults in Disadvantaged Areas in Sweden

**DOI:** 10.1093/geroni/igaf122.1783

**Published:** 2025-12-31

**Authors:** Marianne Granbom, Afsaneh Taei, Anders Kottorp

**Affiliations:** Lund University, Lund, Skane Lan, Sweden; Lund University, Lund, Skane Lan, Sweden; Malmo University, Malmo, Skane Lan, Sweden

## Abstract

Disadvantaged residential areas (DAs), often affected by territorial stigmatization, influence the health and quality of life in older adults. Nevertheless, older adults may prefer to stay in these areas due to emotional bonds. With the Person-Place Fit Measure for Older Adults (PPFM-OA), older adults self-assess how their home and community environments suit their needs. The aim of the study was to explore associations between person-place fit and quality of life among older adults living in different types of DAs. Survey data were collected from 459 older adults living in urban areas with low socioeconomic status and depopulated rural areas in Sweden. Person-place fit was measured using the Swedish 19-item version of the PPFM-OA and quality of life was assessed with WHOQOL-BREF. Mean age was 76 years (SD 7), with 51% being women and 13% were not born in Sweden. Linear regression models demonstrated significant associations between person-place fit and all aspects of quality of life (overall QoL, overall health, physical health, psychological health, social relationships, and environment) even after adjusting for sex, gender and ADL performance. No differences were found between residents of urban and rural DAs. The study shows that older adults’ perceptions of how well their home and community environments meet their needs are associated with their quality of life. Furthermore, the results suggest that residential areas that, from an outsider perspective are considered non-age-friendly, may not be perceived as such by older the older residents.

